# Consolidating food safety measures against COVID-19

**DOI:** 10.1186/s42506-022-00112-6

**Published:** 2022-11-02

**Authors:** Assem Abolmaaty, Dina H. Amin, Reham M. M. Abd El-kader, Alaa F. ELsayed, Basma S. M. Soliman, Amr S. Elbahnasawy, Mahmoud Sitohy

**Affiliations:** 1 Department of Food Science, Faculty of Agriculture, Ain Shams University, Cairo, Egypt; 2 Department of Microbiology, Faculty of Science, Ain Shams University, Cairo, 1566 Egypt; 3 Radiation Microbiology Department, National Center for Radiation Research and Technology, Egyptian Atomic Energy Authority, Cairo, Egypt; 4 Department of Biochemistry and Nutrition, National Food Safety Authority, Cairo, Egypt; 5 Department of Bioecology, Hygiene and Public Health, Institute of Fundamental Medicine and Biology, Kazan Federal University, Kazan, Russia; 6 Department of Nutrition and Food Sciences, National Research Centre, Giza, Egypt; 7 Department of Biochemistry, Faculty of Agriculture, Zagazig University, Zagazig, 44511 Egypt

**Keywords:** COVID-19, Food environment, Food safety, Good hygiene practice

## Abstract

**Background:**

The world is facing an extraordinarily unprecedented threat from the COVID-19 pandemic triggered by the SARS-CoV-2 virus. Global life has turned upside down, and that several countries closed their borders, simultaneously with the blockage of life cycle as a result of the shutdown of the majority of workplaces except the food stores and some few industries.

**Main body:**

In this review, we are casting light on the nature of COVID-19 infection and spread, the persistence of SARS-CoV-2 virus in food products, and revealing the threats arising from the transmission of COVID-19 in food environment between stakeholders and even customers. Furthermore, we are exploring and identifying some practical aspects that must be followed to minimize infection and maintain a safe food environment. We also present and discuss some World Health Organization (WHO) guidelines-based regulations in food safety codes, destined to sustain the health safety of all professionals working in the food industry under this current pandemic.

**Conclusion:**

The information compiled in this manuscript is supporting and consolidating the safety attributes in food environment, for a prospective positive impact on consumer confidence in food safety and the citizens’ public health in society. Some research is suggested on evaluating the use and potentiality of native and chemical modified basic proteins as possible practices aiming at protecting food from bacterial and viral contamination including COVID-19.

## Background

Coronaviruses are affiliated to the Coronaviridae family under the order Nidovirales. The word corona refers to crown-like spikes on the outer virus surface. Coronaviruses are a single-stranded RNA with tiny molecular size, 65–125 nm in diameter, and 26 to 32 kbs in length [1]. They are infectious to birds and several vertebrates, including humans, causing respiratory tract and gastrointestinal tract infections [2]. The severe acute respiratory syndrome coronavirus (SARS-CoV), H5N1 influenza A, H1N1 2009, and Middle East respiratory syndrome coronavirus (MERS-CoV) were globally reported to trigger acute lung injury (ALI) and acute respiratory distress syndrome (ARDS), causing pulmonary failure which leads finally to death [3].

A severe acute respiratory syndrome (SARS) outbreak was recently reported by SARS-CoV, 2002, in Guangdong, China [1]. A decade after, another dangerous infectious coronavirus, i.e., Middle East respiratory syndrome coronavirus (MERS-CoV), erupted in the Middle Eastern countries leading to another endemic [4]. Most recently, COVID-19 has lately outbroken at the end of 2019, killing above eighteen hundred and infecting more than seventy thousand individuals during the first 50 days of its epidemic emergence in a business center of China, Wuhan [5, 6]. The outbreak of this obscure pneumonia in Wuhan city was associated with food, since most of the patients were reported to have visited the Huanan Seafood Wholesale Market [7]. The Chinese researchers called the novel virus Wuhan coronavirus or 2019 novel coronavirus (2019-nCov). Alternatively, the International Committee on Taxonomy of Viruses (ICTV) named the virus as SARS-CoV-2 and the disease as Covid-19. The COVID-19 is still a highly contagious disease, reporting over 23 million infection cases and harvesting over 800,000 death cases [5, 6].

Highly efficacious transmission of SARS-CoV-2 between human, globally experienced, is creating probably unprecedented pandemic at least for the last two centuries [7–9]. The widespread global travel activities and the ease of use could further boost this worldwide spread [10]. The interconnected international food trade and mutual dependence worldwide can create another threat if not well scrutinized and controlled.

## Symptoms of COVID-19

It was reported that patients confirmed with COVID-19 mainly had respiratory signs and symptoms [11, 12]. In most cases, fever was reported, associated with a dry cough [13]. Vague gastrointestinal symptoms such as nausea, vomiting, and diarrhea were notably reported from a significant proportion of COVID-19 patients [11]. Myalgia or fatigue was also reported [13]. In contrast to human previously experienced coronavirus infections, upper respiratory symptoms are remarkably infrequent in COVID-19 patients [14]. A new onset of COVID-19 infection-associated psychosis potentially connected with immune-mediated neuropathogenesis was recently reported [15]. The viral load of SARS-CoV-2 reaches its peak within 5–6 days of symptom onset, i.e., significantly much quicker than SARS-CoV, which requires about 10 days. Severe COVID-19 cases can progress into acute respiratory distress syndrome (ARDS) [7].

## Route of transmission, pathophysiology, and testing of SARS-CoV-2 infection

Coronaviruses are known viruses causing disease in humans and animals. Most strains uniquely infect the upper respiratory tract and cause mild common cold symptoms [16]. However, three coronaviruses, namely, severe acute respiratory syndrome coronavirus (SARS-CoV), Middle East respiratory syndrome coronavirus (MERS-CoV), and SARS-CoV-2, can replicate in the lower respiratory tract, causing probably fatal pneumonia. SARS-CoV-2 belongs to the betacoronavirus genus. It is closely related to SARS-CoV, with 79% genetic similarity [17]. The species severe acute respiratory syndrome-related coronavirus is classifying 2019-nCoV and naming it SARS-CoV-2. Like other respiratory coronaviruses, SARS-CoV-2 is transmitted predominantly through droplets. Droplet transmission happens when a person comes into close contact (within 1 m) with someone who has respiratory symptoms (e.g., coughing or sneezing) and is therefore exposed to potentially infective respiratory droplets on his or her mucosae (mouth and nose) or conjunctiva (eyes). Transmission can also happen through fomites in the sick person’s immediate environment [18]. After infection, the average incubation period is usually 4–5 days before symptoms appear; around 97% of symptomatic patients have manifestations in the first 10 days of exposure to the virus [19]. Based on the available information, there is no proof to propose the transmission of COVID-19 via fecal-oral route. However, it was reported that some stool samples of infected patients contained the RNA (ribonucleic acid) of COVID-19 might cause the infection [11, 20]. Clear evidence reported that the transmission of COVID-19 virus is due to droplets containing viable virus landed on any surface. It can be transmitted by self-inoculation via mucous membranes of the nose, eyes, and mouth [21–23]. Remarkably, about 108 viral copies were detected in only 1 ml of the infected person’s sputum [23, 24]. Consequently, the whole environment could be a source of infection via direct or indirect contact transmission [25–27]. Animals are suspected vectors of COVID-19 virus and are expected to be the cause of original infection and the current outbreak of epidemy, as confirmed by European Center for Disease Prevention and Control (ECDC).

SARS-CoV-2 infection’s pathophysiology is similar to SARS-CoV but with stronger inflammation reactions that can end by airways damage, depending on various host and virus factors that determine COVID-19 disease severity in patients. Old age and underlying chronic diseases are major host factors correlated to the disease severity [27]. Acute respiratory distress syndrome in severe COVID-19 can range from breathing difficulties, low blood oxygen level, and up to respiratory failure, which is the major death cause in 70% of COVID-19 deaths [28]. Also, cytokines storm in response to the viral infection together with associated secondary bacterial infections can lead to septic shock causing death in 28% of COVID-19 deaths. Most deadly cases manifested multiorgan failure due to uncontrolled inflammatory reactions particularly in cardiac, hepatic, and renal systems [29].

To confirm diagnosis of SARS-CoV-2 infection, direct virological tests mainly molecular diagnosis must be done to all suspected cases. Suspected cases are those with respiratory symptoms likely to be SARS-CoV-2 or healthcare workers with repeated risk of exposure to the virus. Reverse transcriptase-polymerase chain reaction tests are highly recommended [30]; however, other recent tests have emerged. Nasopharyngeal samples are best samples used to detect SARS-CoV-2 according to the CDC (Centers for Disease Control and Prevention) [31]. Nasal swabs or oropharyngeal swabs are also accepted. Lower respiratory tract samples have higher sensitivity than upper tract samples, but they are not used to avoid virus aerosols and spreading of the virus during sample collection [32].

Serological testing to detect viral antibodies indicates the patient was recently or previously infected with SARS-CoV-2. So, serological tests cannot be used alone to diagnose COVID-19 infection; moreover, it may take up to 21 days after symptom onset for seroconversion to detect immunoglobulin M and/or immunoglobulin G antibodies to SARS-CoV-2 [33]. Finally, the sensitivity and specificity of many commercially available serologic tests are not high. For example, false-positive test results can occur due to possible antibodies cross-reactivity with other coronaviruses strains. It is still unknown how long antibodies can persist after infection and whether the presence of antibody reflects protective immunity against future infections [34].

## Hazards triggering COVID-19 virus transmission in food environments

**Fig. 1:**
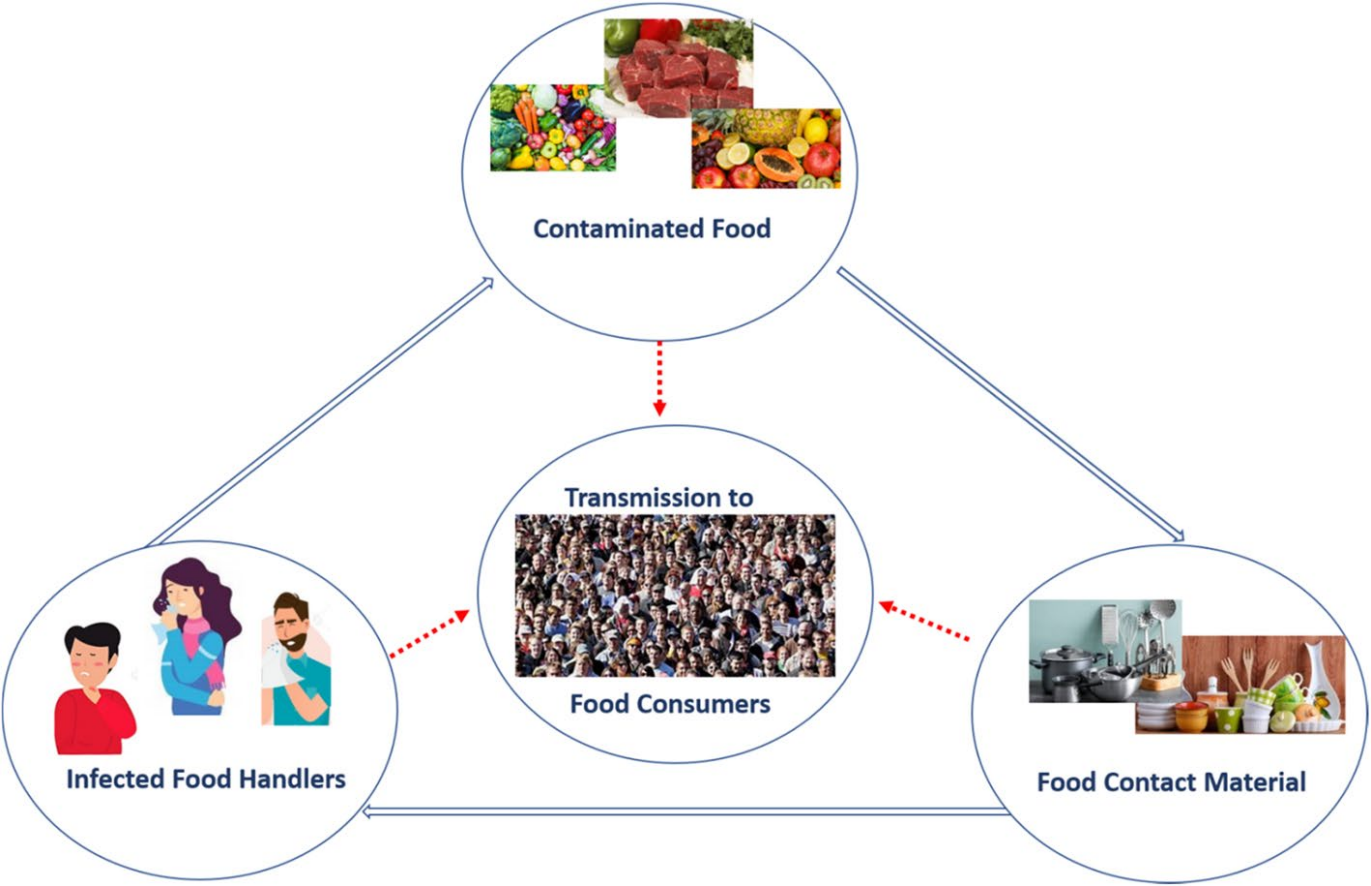
The potential transmission pathway of coronaviruses via food products and processes [35]

Three main hazards can cause the spread of the COVID-19 virus in food environments, including contaminated food, infected food handlers, and contaminated food contact materials [35], as shown in Fig. 1.

The European Food Safety Authority (EFSA) declared that no confirmation is indicating food as a possible source of transmission of the virus (COVID-19) [36]. This conclusion is based on previous knowledge regarding the nature of other similar viruses such as SARS-CoV and MERS-CoV, assuming their similar route of transmission. This may explain the absence of the European Food Safety Authority (EFSA) participation in response to COVID-19.

It was reported that the threat of coronavirus infection depends on some factors, including the type of contaminated surfaces, the amount of virus, and the time of getting contact with the virus [37]. In order to stop the spread of COVID-19 infection, good hygiene practices should be extremely applied [26, 38].

Object exposure is a possible route for COVID-19 transmission [32]; it includes an inanimate object carrying a pathogen from one susceptible person to another, through touching the surface and then the eyes, nose, or mouth. The virus can remain viable and infectious for hours in air and for days on surfaces [39]. Viral stability of human coronaviruses, SARS-CoV-1 and SARS-CoV-2, was examined in aerosols and on various surfaces, i.e., plastic, stainless steel, copper, and cardboard [39, 40]. The coronavirus’s viability in the materials above was 3, 72, 72, 4, and 24 h, respectively [39]. Some information refers that food handlers in meat and poultry factories may be the primary source of indoor bioaerosols in the food industry via spreading microorganisms through contact, coughing, or sneezing and by their clothes [41].

Currently, there is no evidence suggesting that food or food packaging could be a possible route for COVID-19 transference to humans, or that humans can contaminate animal food. Product packaging should be taken out and left for air-drying as an additional precaution [42].

## Persistence of coronavirus on food products

Human coronaviruses can persist on solid surfaces at room temperature for up to 9 days. However, at a temperature equal to or above 30 °C, they persist for a shorter duration. On the other hand, veterinary coronaviruses were shown to remain infectious on surfaces up to 28 days [23]. A recent review of human coronaviruses’ persistence on surfaces reported large variability, ranging from 2 h to 9 days [23]. The duration that the virus during which COVID-19 can persist on surfaces is not yet fully confirmed. However, it seems mostly to act like other coronaviruses.

Some viruses like avian influenza viruses and coronaviruses have the potential for foodborne transmissions. Foodborne viruses can survive for extended periods at low pH values from 3 to 4 and at high pH from 9 to 10 [43]. Until now, there are no reports on the transmission of SARS-CoV-2 by food. Studies to estimate the mode and time of virus survival in food matrices are in progress. For example, MERS-CoV has been demonstrated to survive up to 72 h in food at 4 °C [44].

Since COVID-19 virus possesses a fragile outer membrane, it is considered one of the enveloped viruses that are less stable in the environment and are more sensitive to oxidants, like chlorine. There is no confirmation about the survival of the COVID-19 virus in water or sewage. Enveloped viruses are expected to become highly inactivated and faster than the non-enveloped human enteric viruses which are known to be transmitted by water (such as adenoviruses, norovirus, rotavirus, and hepatitis A). As an example, a study found that an alternate human coronavirus survived just 2 days in dechlorinated tap water and in hospital wastewater at 20 °C [45]. Human coronaviruses, transmissible gastroenteritis coronavirus, and mouse hepatitis virus showed a 99.9% die-off from 2 days at 23 °C [46] and 2 weeks at 25 °C [47]. Heat, high or low pH, sunlight, and common disinfectants (such as chlorine) all speed up the virus die-off.

“Recent research has assessed the COVID-19 pathogen's ability to survive on various surfaces.” They have demonstrated that the virus can survive on plastic and stainless steel for up to 72 h, on copper for up to 4 h, and on cardboard for up to 24 h. The relative humidity and temperature were controlled in a laboratory setting for this investigation; thus, results should be taken with care in relation to actual settings [18].

## Potential inactivation of coronaviruses on food contact surfaces

It is recommended by the World Health Organization (WHO) to safeguard all environmental surfaces by routine cleaning and disinfection procedures. Standard sanitization using water and detergents must be used to disinfect the viral loads from any surface throughout food manufacturing, packaging, and supply processes [38, 48]. Transmission of coronaviruses from contaminated surfaces to hands is not still wholly assured [23], while a transmission rate of 31.6% was confirmed for the influenza A virus, enabling the virus to be transmitted to hands within 5 s from contacting contaminated surfaces [49]. Human coronavirus (HCoV) strain 229E remains infectious for a period ranging from 2 h to 9 days on diverse kinds of materials. In case of (MERS) coronavirus, shorter persistence was recorded at elevated temperatures (30 °C or 40 °C) than lower ones. In terms of viral concentration, higher viral loads of SARS-CoV recorded longer persistence than lower ones. Higher relative humidity boosted the persistence of HCoV-229E than lower ones [23, 50]. In this review, we will focus on different probable ways to disinfect the viral loads on food products and related environments. We also shed light on control measures that affect similar viruses and therefore open the door for future research on COVID-19, as shown in Table 1 [23, 51].

**Table 1:** Potential measures to disinfect the viral loads on food products and related environment

Inactivation procedure	Sample type	Condition	Virus type	References
**Heat treatments**	Environmental samples	90 min at 56 °C 60 min at 67 °C 30 min at 75 °C	SARS coronavirus	[51]
**UV irradiation**	Environmental samples	60 min	SARS coronavirus	[51]
**Chemical treatments (ethanol 62–71%)**	Small surfaces Carrier test	1 min	Human coronaviruses (HCoV)	[23]
**Sodium hypochlorite concentration 0.1%**	Large surfaces	1 min	Human coronaviruses (HCoV)	[23]
**Hydrogen peroxide 0.5%**^a^	Surfaces	1 min	Human coronaviruses (HCoV)	[23, 51]

^a^ECDC technical report, interim guidance for environmental cleaning in non-healthcare facilities exposed to SARS-CoV-2 (2020)

## Recommended hygiene practices for preventing the transmission of coronaviruses

The World Health Organization (WHO) advised the nations for physical separation and close working places. However, some industries and individuals are mandatorily still working in their normal workplaces. It has also issued some safety measures throughout the process of food handling and preparation. All food workers should safeguard the obligatory personal and environmental controls and Hazard Analysis Critical Control Point (HACCP) regulations to prevent the transmission of COVID-19 [44]. Implementation of food safety codes and good hygiene practices during food manufacturing, packaging, and supply processes decrease enormously the risk of contamination from food contact materials [44, 52].

In order to eliminate or reduce the risk of viral contamination on the surface of food products and packaging materials. When used appropriately, personal protective equipment (PPE) like masks and gloves can effectively stop the spread of viruses and diseases in the food sector. Additionally, the food industry is urged to keep a safe physical distance, implement stringent hygiene and sanitation procedures, and support frequent and efficient handwashing and sanitation at every stage of food processing, production, and marketing. These precautions will safeguard workers from the COVID-19 virus, keep the workforce healthy, and allow for the detection and ejection of infected employees and others in close contact with them [53].

Food workers include food manufacturers and catering workers who directly relate to unpacked food products due to their work. This category also includes personnel who may touch surfaces in contact with food or other surfaces in rooms where unpackaged food is produced. Thus, this term can be applied to managers, cleaners, maintenance contractors, suppliers, and food inspectors [54]. Food industry personnel should be aware of the symptoms of COVID-19. Food industry operators should prepare written recommendations for employees on reporting the symptoms of the disease and the suspension policy. The most important problem is employees’ ability to recognize symptoms at an early stage of the disease so that they can seek appropriate medical care, testing, and minimize the risk of spreading the virus [54].

The proper handwashing with soap and hot water for at least 20 s is considered acceptable hygiene practices by staff (as recommended by World Health Organization) [44]. It is also a good idea to frequently use alcohol-based hand sanitizers and to breathe properly (cover your mouth and nose when you cough or sneeze). Wipe disposal, routine cleaning, and disinfection of work areas and potential contact points, such as door handles, are also advised. Avoiding close contact with people who are coughing or sneezing is also a good idea [55]. Food workers can use gloves, but they must be often changed, and hands should be washed whenever gloves are removed or change gloves to avoid subsequent food contamination [44]. Gloves must be changed after nonfood-related activities, such as manually opening/closing doors and emptying trash cans. When wearing gloves, food workers should not touch their mouth and eyes, since the COVID-19 virus can contaminate disposable gloves. Wearing disposable gloves can create a false sense of security, leading personnel to wash their hands less often than necessary. Then, handwashing is a more serious protective barrier to infection than wearing disposable gloves. It is crucial to remind that gloves do not provide complete protection against contamination with patients’ flora; thus, hands must still be decontaminated after wearing them [56]. And if not used properly, gloves might contribute to the spread of infections [57]. Food industry enterprises must ensure proper sanitation and instruct staff to wash their hands thoroughly and often. Hand sanitizers can be used as an additional measure, but should not replace handwashing [58].

Keeping a physical distance to minimize the contact between potentially infected and healthy people is an important measure to slow down the quick spread of COVID-19. All food processing companies should follow physical distance guidelines as far as possible. The World Health Organization recommendations necessitate maintaining at least 1-m distance between employees [44]. In cases where the food production environment or process makes it difficult to comply with this recommendation, employers should consider possible measures to protect workers such as staggering jobs on both sides of production lines so that employees are not facing each other. The use of personal protective equipment (PPE) should become standard practice in high-risk areas in the premises of enterprises where ready-to-eat foods are produced.

## Transportation and delivery of food raw materials and food products

The main attention to meet the food industry’s sanitary and hygienic requirements is paid to prevent the entrance and spread of the COVID-19 virus into the food enterprises. A virus can only enter office premises if an infected person enters the premises or brings in infected products or objects. Drivers and other food delivery personnel must not leave vehicles at the time of delivery. Drivers should be provided with alcohol-based hand antiseptics, disinfectants, and paper towels. Disposable containers and packaging should be used to avoid the need for disinfection of any returns. If reusable containers are used, appropriate hygiene protocols must be followed [38]. Drivers delivering food to premises should be aware of the potential risks associated with COVID-19 contact transmission. The virus can be transmitted by contact with an infected surface or shaking hands with an infected person. Surfaces that are more likely to be affected by the virus include surfaces that are most often touched, such as the steering wheel of a vehicle, door handles, and mobile devices. Therefore, hand hygiene combined with physical distance is of paramount importance along with disinfection contact surfaces to prevent cross-contamination [38].

The risk of transmitting COVID-19 should be reduced by identifying surfaces and objects most often touched in retail spaces and ensuring regular cleaning and disinfection. Examples of such surfaces and objects are shopping trolleys and baskets, door handles, and scales for customers [38]. Providing napkins (or other disinfectants) for cleaning the handles of carts and baskets; or the appointment of staff to disinfect the handles of carts and baskets after each use, cleaning and frequent disinfection of accessories used in the store (scoops, tongs, left-luggage offices), open doors if possible, to reduce contact open counters and shelves in retail stores [55].

Although some consumers believe that there is a risk of getting COVID-19 through open food stalls and shelves, there is currently no scientific evidence that food can be a virus transmission source. However, it is essential to maintain acceptable hygiene standards near open counters and shelves, such as salad bars, shelves with fresh produce, and bakery products [38]. Consumers are always advised to wash their fruits and vegetables before eating. Customers and store staff must strictly observe personal hygiene at any time near open counters and shelves. Self-service in stores should be laid out in plastic/cellophane or paper packaging. For displaying bakery products in retail stores, plexiglass display cases should be used, and each product should be placed in a separate package using forceps when serving customers.

## Food irradiation as a possible solution to fight COVID-19

Irradiation effect on viruses is inversely proportional to its structural complexity. Large-sized viruses are more sensitive to ionized radiation than the smaller ones. Viruses require higher doses of irradiation than bacteria and fungi because of their minute sizes. However, viruses with double-stranded nucleic acid (dsRNA and dsDNA) are more resistant to UV irradiation than single-stranded nucleic acid (ssRNA and ssDNA). The lethal effect of ionizing radiation on viruses increases in the presence of oxygen. Viruses irradiated in a liquid medium are more sensitive than in dried or frozen samples [59].

The mechanism of radiation for viral inactivation occurs by the destruction of replicating nucleic acid by two ways, either directly through radiolytic cleavage and cross-linking the genetic material or indirectly via free radicals on nucleic acids and proteins [60]. Using cobalt-60 as a source of gamma irradiation is in general an efficacious and safe laboratory way for the inactivation of the infection specimen. The Centers for Disease Control and Prevention (CDC) have used a high dose (2 × 10^6^ rad) to inactivate SARS-CoV-infected serum specimens [61]. A research group also deduced that the same range of dose 3–4.5 × 10^6^ rad is needed to inactivate viruses in monoclonal antibody preparations [62]. The D10 range for viruses in the Coronaviridae family is estimated to be up to 3.6 kGy [63].

Another study revealed that the viruses were completely inactivated by a 1 M rad irradiation dose on SARS-COV [64]. The absorbed dose of gamma irradiation depends on many factors such as sample volume, sample origin, sample composition, irradiation temperature, distance to irradiation source, and others [60]. Two Kev (kilo-electronvolts) irradiation electron energy from electron beam irradiation was required to interact with Covid-19 virus for the highest energy loss and complete activity destruction based on the gene sequence collected from Wuhan patients [65].

UV radiation was categorized into three levels (AVA, UVB, and UVC) based on their influence on SAR-CoV toxicity: 320-400, 280-320, and 200-280 nm, respectively. UVA is weakly absorbed by DNA and RNA and is less effective than UVC and UVB in inducing pyrimidine dimers. However, additional genetic damage may occur through the production or reactive oxygen species, which cause oxidization of bases and strand breaks [66]. UVB may induce pyrimidine dimers but less efficiently than UVC by 20- to 100-fold [67]. RNA and DNA bases can absorb UVC, causing the photochemical fusion of two adjacent pyrimidines into covalently linked dimers and rendering them non-pairing bases [67]. Illumination with different wavelengths also influenced activities of SARS and MERS virus in blood: UVA [68] and UV-B light [69]. These commercial systems could reduce SARS and MERS virus activities in plasma or platelet concentrates to different degrees. Methylene blue plus visible light was also found able of inactivating coronaviruses in plasma [70].

UV-C lamps could be utilized to supplement existing hospital cleaning and disinfection of SARS-CoV-2-infected surfaces as reported by Lualdi et al. (2021). When it comes to choosing, setting, and confirming UV-C lights, there are a few simple guidelines to follow. Photochromic UV-C dosimeters may be a valuable tool for readily checking that an appropriate UV-C dose has been supplied in the absence of devices specialized to direct irradiance verification [71].

## Basic proteins as a possible surface treatment against foodborne COVID-19 virus

**Fig. 2:**
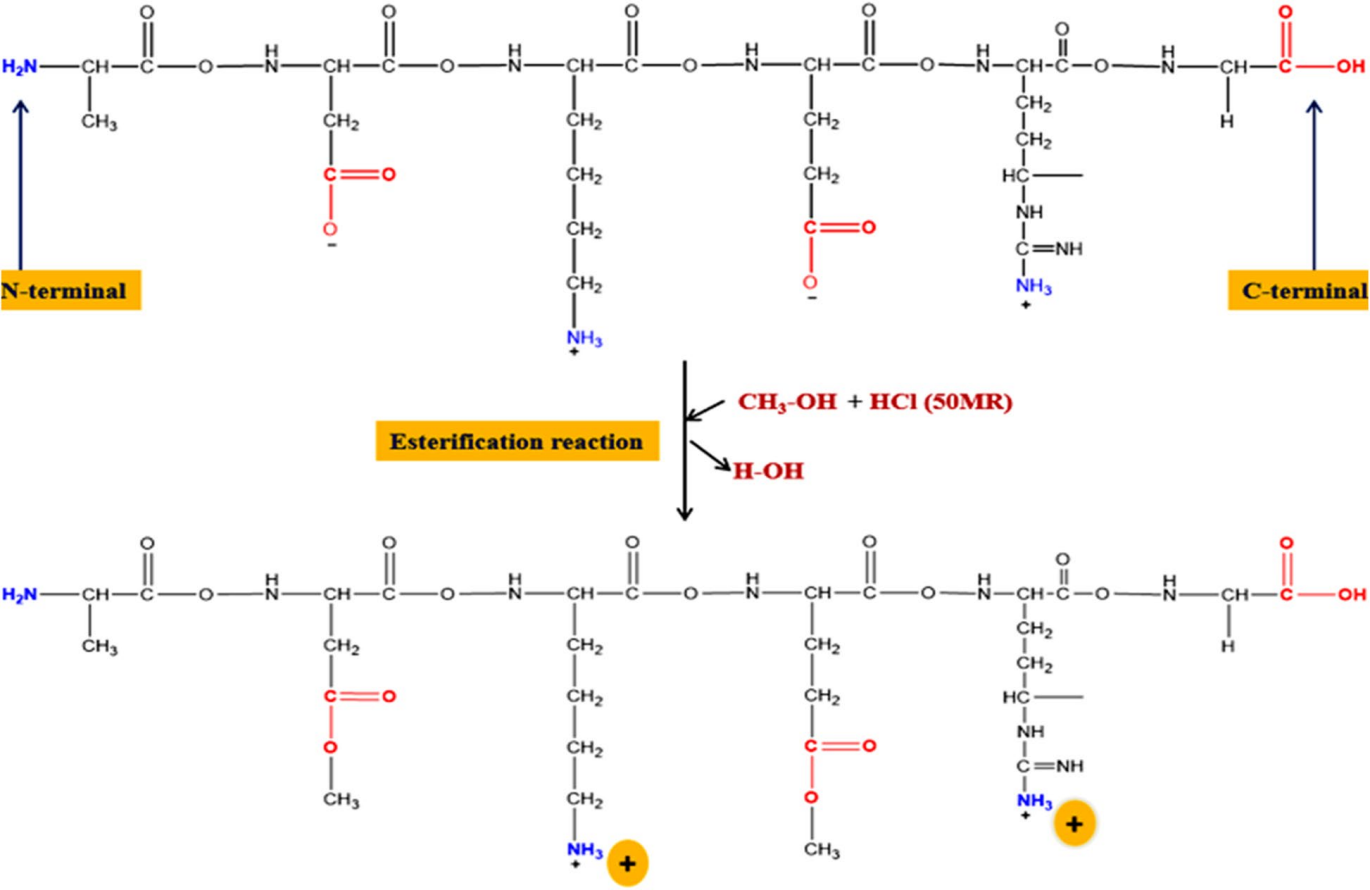
Esterification reaction [88]

**Fig. 3:**
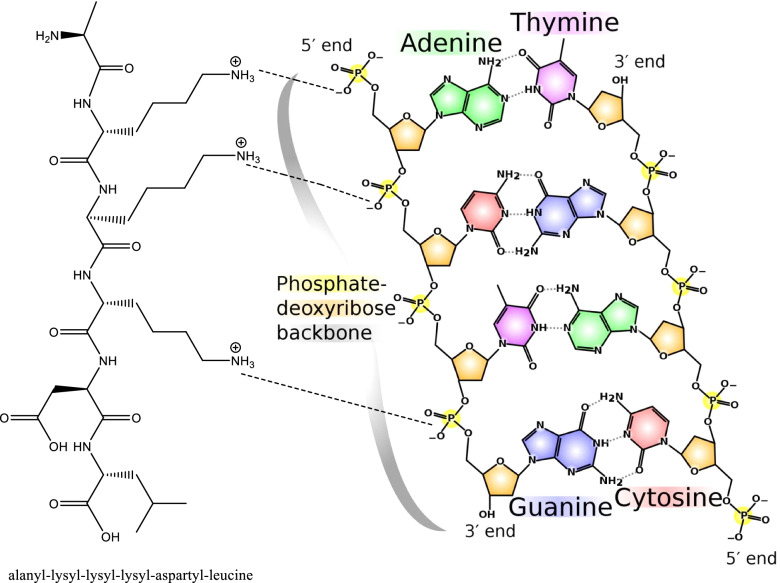
Potential electrostatic interactions between basic proteins and nucleic acids (DNA and RNA) [89]

Basic proteins, either native or chemically modified, were reported as both wide-spectral antibacterial [72–75] and nonspecific antiviral active substances [76–82]. This dual activity can be largely invested in food protection and preservation based on previous results [74, 83–87] as food may be contaminated by both bacteria and virus. Chemically modified proteins can have their carboxyl groups (on their aspartyl/glutamyl residues) neutralized by grafting the alcohol groups during esterification on protein molecules, transforming the protein net charge into positive, and becoming cationic proteins with also enhanced hydrophobicity coming from the grafted alkyl groups as shown in Fig. 2 and Fig. 3 [88, 90]. These positive charges enable them to chemically complex and hence inactivate naked DNA or RNA [89, 91, 92].

The main mechanism of the cationic of action against bacteria is through disturbing the cell membrane and cell wall structures, mainly through targeting the negatively charged components in the cell membrane, i.e., phospholipids by the positively charged proteins [93–97]. Alternatively, the mechanism of the antiviral action is envisaged to occur during viral replication where the virus is released from its coat, and thus, the negative charge on the phosphate backbone of viral DNA or RNA is disclosed and becoming subjected and liable to the electrophilic attack of the positively charged basic proteins [98, 99]. So, these basic proteins can attack any virus if it could exist in site during the phase of replication. Since virus is multiplying rapidly and thus disclosing negatively charged fragments, it will be an easy target for the basic protein action. The ability of these proteins to complex DNA may disturb its functional pathways particularly during replication. A thick coat of proteins usually protects viral DNA or RNA, but it is totally bare at the moment of replication. Therefore, the success of this strategy depends on the availability of viral DNA or RNA in the naked status during the replication process.

Lactoferrin is a native cationic protein with nonspecific antiviral activity [100]. Native basic protein, lactoferrin, was found effective against plant viruses [100]. Moreover, lactoferrin was even reported active against COVID-19 in human being, and that it is commercially available for different medical application mainly as antibacterial or antiviral under commercial formulas. More recently, this was reported as a direct remedy for COVID-19 based in a medical case study on 75 COVID-19 patients [101] when administered as liposomal bovine lactoferrin (LLF) nutritional syrup food supplement (32 mg of LF/10 ml plus 12 mg of vitamin C). As the same treatment at lower dose prevented the disease in healthy persons, it may prospectively be used in food protection against contamination with that virus. This may also refer to the potentiality of other cationic proteins either native or chemically modified to act the same function.

Globally, these results suggest a wide-spectrum specificity of these chemically modified proteins against different viruses and pathogenic bacteria nominating them as potential effective candidate in treating food products as a precautionary measure against contamination with COVID-19.

## Conclusion

This review highlights the food security guidelines aiding at overcoming the current pandemic peacefully. Generally, the most important precaution is to keep the COVID-19 virus or other viruses out of the food environment which is in continuous contact with every human being. Future preventive intervention may invest some bioactive natural products, lactoferrin, and chemically modified proteins in cleaning and keeping fresh food products out of the reach of viral contamination. Key measures are required by upgrading the cleaning and sanitation measures, disinfecting surfaces, and educating staff on the virus and how to protect themselves and others. Combined protocols such as handwashing, physical distancing, sanitizing hands, and other material should be added and improved. Generally, new ways and means should be investigated to counteract potential food contamination. Specifically, it is urgently needed to evaluate the use and potentiality of native and chemical modified basic proteins as possible practices for protecting food from bacterial and viral contamination including COVID-19.

## Data Availability

Not applicable

## Abbreviations

ALI: Acute lung injury
ARDS: Acute respiratory distress syndrome
CDC: Centers for Disease Control and Prevention
dsRNA and dsDNA: Double-stranded nucleic acid
ECDC: European Center for Disease Prevention and Control
EFSA: The European Food Safety Authority
FDA: Food and Drug Administration
HACCP: Hazard Analysis Critical Control Point
HCoV: Human coronavirus
ICTV: International Committee on Taxonomy of Viruses
Kev: Kilo-electronvolts
LLF: Liposomal bovine lactoferrin
MERS-CoV: Middle East respiratory syndrome coronavirus
PPE: Personal protective equipment
RNA: Ribonucleic acid
SARS-CoV: Severe acute respiratory syndrome coronavirus
SARS: Severe acute respiratory syndrome
ssRNA and ssDNA): Single-stranded nucleic acid
WHO: World Health Organization
2019-nCov: 2019 novel coronavirus
