# Correction: Consolidating food safety measures against COVID-19: a review

**DOI:** 10.1186/s42506-023-00137-5

**Published:** 2023-07-06

**Authors:** Assem Abolmaaty, Dina H. Amin, Reham M. M. Abd El‑kader, Alaa F. ELsayed, Basma S. M. Soliman, Amr S. Elbahnasawy, Mahmoud Sitohy

**Affiliations:** 1 Department of Food Science, Faculty of Agriculture, Ain Shams University, Cairo, Egypt; 2 Department of Microbiology, Faculty of Science, Ain Shams University, Cairo, 1566 Egypt; 3 Radiation Microbiology Department, National Center for Radiation Research and Technology, Egyptian Atomic Energy Authority, Cairo, Egypt; 4 Department of Biochemistry and Nutrition, National Food Safety Authority, Cairo, Egypt; 5 Department of Bioecology, Hygiene and Public Health, Institute of Fundamental Medicine and Biology, Kazan Federal University, Kazan, Russia; 6 Department of Nutrition and Food Sciences, National Research Centre, Giza, Egypt; 7 Department of Biochemistry, Faculty of Agriculture, Zagazig University, Zagazig, 44511 Egypt

**Correction: J Egypt Public Health Assoc 97, 21 (2022)**

**https://doi.org/10.1186/s42506-022-00112-6**

Following publication of the original article [1], we have been notified that the title of the article was published incorrectly. It should be as follows:

Consolidating food safety measures against COVID-19: a review

The original article [1] was updated.
